# Using Facebook for Health Promotion in “Hard-to-Reach” Truck Drivers: Qualitative Analysis

**DOI:** 10.2196/jmir.9689

**Published:** 2018-11-01

**Authors:** Marguerite C Sendall, Laura K McCosker, Phil Crane, Bevan Rowland, Marylou Fleming, Herbert C Biggs

**Affiliations:** 1 School of Public Health and Social Work Faculty of Health Queensland University of Technology Brisbane Australia; 2 School of Social Sciences Faculty of Arts, Business and Law University of the Sunshine Coast Sippy Downs Australia; 3 Centre for Accident Research and Road Safety - Queensland Queensland University of Technology Brisbane Australia

**Keywords:** communication, health promotion interventions, mobile phone, social media, transport industry, truck drivers, workplace health promotion, workplace managers

## Abstract

**Background:**

Workers in the road transport industry, and particularly truck drivers, are at increased risk of chronic diseases. Innovative health promotion strategies involving technologies such as social media may engage this “hard-to-reach” group. There is a paucity of evidence for the efficacy of social media technologies for health promotion in the Australian transport industry.

**Objective:**

This study analyzed qualitative data from interviews and focus group discussions to evaluate a social media health promotion intervention, the Truckin’ Healthy Facebook webpage, in selected Australian transport industry workplaces.

**Methods:**

We engaged 5 workplace managers and 30 truck drivers from 6 transport industry organizations in developing workplace health promotion strategies, including a social media intervention, within a Participatory Action Research approach. Mixed methods, including a pre- and postintervention manager survey, truck driver survey, key informant semistructured interviews, truck driver focus groups, and focused observation, were used to evaluate the social media intervention. We asked questions about workplace managers’ and truck drivers’ opinions, engagement, and satisfaction with the intervention. This paper focuses on qualitative data.

**Results:**

Of the workplace managers who reported implementing the social media intervention at their workplace, all (3/3, 100%) reported satisfaction with the intervention and expressed a keen interest in learning more about social media and how it may be used for workplace health promotion and other purposes. Truck drivers were poorly engaged with the intervention because (1) many believed they were the “wrong age” and lacked the necessary skills; (2) the cost of smartphone technology was prohibitive; (3) they confined their use of social media to nonwork-related purposes; and (4) many workplaces had “no Facebook” policies.

**Conclusions:**

The use of social media as a health promotion intervention in transport industry workplaces has potential. Workplace interventions using social media can benefit from a Participatory Action Research approach. Involving managers and workers in the design of social media health promotion interventions and developing strategies to support and deliver the interventions helps to facilitate their success. The workers’ profile, including their age and familiarity with social media, and work, workplace, and family context is important to consider in this process. Much more research needs to be undertaken to better understand the effective use of social media to engage “hard-to-reach” groups.

## Introduction

The road transport industry accounts for 2% of Australia’s workforce, employing 192,600 Australians [1,2]. Road transport industry workers, in general, and truck drivers, in particular, are identified at increased risk of chronic diseases such as cardiovascular disease and type 2 diabetes [3,4]. Workplaces are increasingly recognized as environments conducive to health promotion. For truck drivers, their workplace is their vehicle and, as a highly mobile workforce, traditional workplace health promotion strategies may be limited in their effectiveness [5-11]. Innovative new health promotion strategies, including social media technologies, may engage and improve the health outcomes of this “hard-to-reach” group.

The use of social media and related technologies is increasingly popular for personal enjoyment [12], as a business tool [13], and in health promotion interventions [14]. This is true for transport industry workplaces and truck drivers. A 2011 study conducted by a UK trucking magazine found that 52% of surveyed truck drivers used social media in their spare time [15]. A similar survey undertaken in 2016 by a US trucking company reported that 93% of their truck driver respondents used smartphones, and 73% reported they checked social media outlets, such as Facebook, Twitter, and Instagram, at least once each day [16]. In addition, US fleet managers are rapidly adopting smartphones and work-related apps to manage their fleets, with 59% of those surveyed tracking their fleet or managing fleet activities or services through mobile apps [17]. The use of information technologies, such as social media, has become a part of the daily life of modern truckies. While few studies have been conducted in Australia, the popularity of social media within the transport industry internationally suggests it is feasible to use it as a health promotion strategy in the Australian context.

A literature review suggests that truck drivers, in Australia and internationally, use social media and related technologies for a variety of purposes. Facebook is a popular social media platform and has been used by truck drivers for a variety of purposes, including to advocate for improved safety at work, report road accidents, identify and stop fatigued drivers, search for missing drivers, and socially connect with other drivers [18-27]. Other social media technologies are used in the trucking industry to map traffic congestion, identify parts dealers, communicate fatigue laws, plan routes, find truck stops, assist with loading, provide voice-guided navigation, and communicate pickup requirements [28-30].

In Australia, social media have been used in the transport industry for health promotion purposes in a limited capacity. Herbert [31] reported an Australian intervention allowing truck drivers to attend medical appointments by videoconferencing on a smartphone or tablet. Gilson et al [32,33] described smartphone interventions to monitor nutrition and physical activity in Australian truck drivers. Kenny [34] reported an intervention providing counseling and peer support through Facebook and Skype to truck drivers who have been involved in workplace accidents or work-related losses, while an Australian trucking magazine described the use of smartphone apps by drivers to share health and physical activity ideas [35]. Of these interventions, only 2 [32,33] included an evaluation of effectiveness. These studies found that technological barriers with smartphone technology prevented a number of drivers from participating in the intervention [32]; however, positive health behavior change was measured as improved driver self-regulation of healthy choices and was observed in participants [33]. While this suggests potential, with so few studies conducted, there remains a paucity of evidence for the efficacy of social media for health promotion in the Australian road transport industry.

The *Queensland Transport Industry Workplace Health Intervention* was a settings-based, mixed-methods Participatory Action Research (PAR) project. The project identified health promotion interventions for transport industry workplaces to support truck drivers to improve their health knowledge and behavior [36]. Over a two-year period, between July 2012 and June 2014, the project worked with 6 transport industry workplaces in south-east Queensland to develop, implement, and evaluate 7 health promotion interventions. One of these interventions was a Facebook page called *Truckin’ Healthy*, which aimed to communicate physical activity and nutrition health promotion messages to truck drivers. An evaluation of this social media intervention, based on the qualitative data from interviews and focus group discussions, is the focus of this paper.

## Methods

### Approach: Participatory Action Research

The project used a range of methods located within a PAR approach to engage workplaces and, specifically, workplace managers and truck drivers, in the development, implementation, and evaluation of workplace health promotion interventions. PAR is a recognized public health research methodology, which relies on the stakeholders’ involvement in decision making, planning processes, and implementation to produce meaningful change [37,38]. For this project, a mixed-methods framework was utilized with PAR. Truck drivers and workplace managers were engaged in the PAR process through interviews, focus groups, and paper-based evaluation surveys. This paper focuses on the qualitative data from interviews and focus group discussions.

At the commencement of the project in July 2012, workplace managers and truck drivers were invited to participate in interviews and focus groups. These were anonymous, confidential, and audiorecorded with consent. We conducted them at a time and place convenient to the managers or drivers, usually a location at the workplace, such as a manager’s office or tea room. They lasted between 20 and 70 minutes. Interview and focus group questions were open-ended and semistructured, developed by the research team with the aim of encouraging participants to share their experiences, attitudes, and values about workplace health promotion and health promotion interventions.

During the initial PAR process, each of the 5 workplace managers was interviewed, and one focus group was conducted with truck drivers at each workplace (involving approximately 30 truck drivers). We asked questions about the various factors which positively and negatively influence truck drivers’ workplace health behavior and their engagement in health promotion activities. This information was organized into an action plan for each workplace. The action plans were used to prompt workplace managers and truck drivers to provide feedback about interventions proposed by the research team and encourage their own ideas to improve their workplace health behavior and engagement in health promotion activities.

### Social Media Intervention—The Truckin’ Healthy Facebook Page

**Figure 1:**
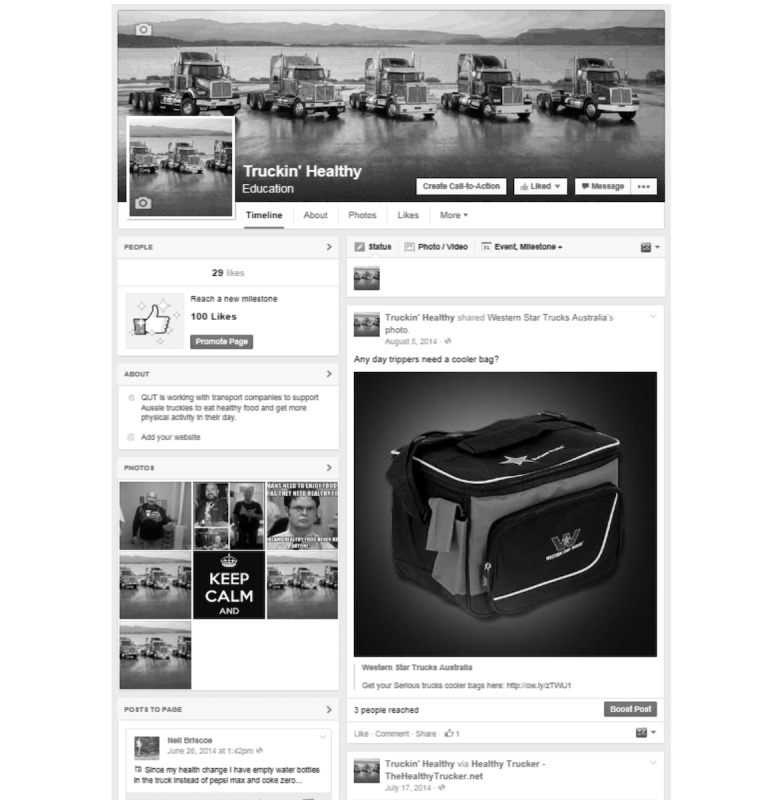
Screenshot of the Truckin’ Healthy Facebook page. Source: Created by the project team at the Queensland University of Technology for the Queensland Transport Industry Workplace Health Intervention.

Through this PAR process, 7 workplace health promotion interventions were developed collaboratively by workplaces and the project team. One of these interventions was the *Truckin’ Healthy* Facebook webpage (Figure 1). During the intervention phase, social media was identified by a number of the workplace managers as a potentially effective way of communicating health promotion messages to their “hard-to-reach” mobile workforce. The *Truckin’ Healthy* Facebook page was developed throughout the intervention period.

**Figure 2:**
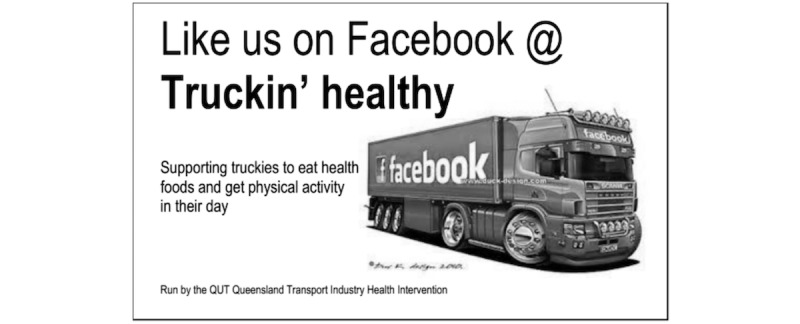
Poster advertising the Truckin’ Healthy Facebook page.

The *Truckin’ Healthy* Facebook page presented content intended to improve participants’ health-related knowledge and behavior. This content included factual health messages, practical exercise tips, simple recipes, health-related videos, and product ideas (eg, lunchbox coolers). Messages were underpinned by the principles of health communication—change-oriented, audience-centered, and situated within a broad ecological perspective. These principle-based messages were integrated with other, multilevel communication strategies and interventions [39]. The information provided was consistent with current national nutrition and physical activity guidelines and delivered in a language targeted to truck drivers. The health-related content was integrated with general interest content related to trucks and the trucking industry. The page was updated at least twice each week throughout the 3-month intervention period. At the end of the intervention period in May 2014, the page was “liked” by 29 people, including truck drivers and 2 transport industry workplace managers from the participating workplaces. Each post had been “shared” or “liked” at least once by page participants.

Workplace managers decided how to promote the *Truckin’ Healthy* Facebook webpage. A poster advertising the page (Figure 2) was developed by the project team, and workplace managers decided where to display the poster in the workplace. Workplaces promoted the Facebook page in other ways. For example, one workplace manager explained:

We [promoted] that on our drivers’ toolbox [talk] as well, for them to go and like it if they wanted to or have a look at it.

TWP120141217

### Data Collection

Data about all the interventions, including the social media intervention, was collected at two time-points— postintervention (3 months after the implementation of interventions commenced) and follow-up (6 months after the implementation of interventions commenced). We collected quantitative and qualitative data. This paper focuses on reporting qualitative data evaluating the social media intervention, with other findings reported elsewhere [40]. At postintervention and follow-up, PAR methods, including semistructured interviews, focus groups, and paper-based evaluation surveys, were used to collect data. Questions were asked about workplace managers’ and truck drivers’ opinions, engagement, and satisfaction with social media intervention.

The project team encountered challenges in facilitating drivers’ and, to a lesser extent, workplace managers’ completion of written surveys. These challenges may have been underpinned by difficulties associated with workplaces prioritizing long-term changes to workplace culture. For this reason, the project team took the approach of collecting the data contained in the surveys using a face-to-face, semistructured interview or focus group format. During postintervention and follow-up, each of the 5 workplace managers participated in at least 1 and up to 2 face-to-face semistructured interviews. A focus group involving approximately 10 truck drivers was conducted at one workplace.

A question in the semistructured interviews, “Did your workplace promote the *Truckin’ Healthy* Facebook page?” produced responses which were rich and textured. Additional questions, including “Did you press ‘like’ to add the *Truckin’ Healthy* Facebook page to your feed?” and “Overall, how satisfied were you with the information provided on the *Truckin’ Healthy* Facebook page?,” produced other detailed responses. Workplace managers commented on the social media intervention when asked questions such as “Which of the initiatives do you consider to be most beneficial for your workplace/employees?” and “What factors made it easy/difficult for you to implement any of the strategies?”

### Data Analysis

A rigorous process of coding-and-theming was used to analyze the data obtained in the interviews and focus groups [41]. After the audiorecordings were transcribed, quotes or significant statements made by truck drivers and workplace managers about the social media intervention were separated from information about other interventions. Using an open and axial coding-and-theming approach [41], these quotes were read several times and then organized into groups of ideas by one member of the project team. This process was repeated until each of the quotes settled into a discrete theme. These themes were then discussed critically, and agreed upon, with other project team members. These themes represent the key aspects of drivers’, workplace managers’, and workplaces’ engagement with social media as a health promotion intervention. In this study, 6 themes were identified.

### Ethical Considerations

This research was approved by the Queensland University of Technology Human Research Ethics Committee (approval number 1300000412).

### Trustworthiness

We applied 4 elements of trustworthiness to this research to ensure rigor [41]. First, the researchers used purposive sampling, ensured privacy and confidentiality, established rapport with participants to create trust and honesty, and asked open-ended questions to ensure authenticity. Next, the sample included a range of truckies (day, pickup and delivery, two-up down, and line haul drivers) to ensure applicability. The research is dependable because the researchers systematically and thoroughly described and documented all phases of data collection and analysis. Finally, to ensure confirmability, all findings are traceable and grounded in the raw data.

## Results

### Overview

In the postintervention interviews, most workplace managers (3/4, 75%) reported they promoted the Facebook page at some time during the intervention period. Of managers whose workplaces participated in this activity, all (3/3, 100%) reported they were “satisfied” with the information contained on the Facebook page, and 2 reported they “liked” the page to add it to their personal Facebook feed. Based on the data provided by workplace managers in the postintervention interviews, of all project interventions, the Facebook page had the second highest rate of implementation and one of the highest rates of satisfaction. However, this did not translate into drivers’ engagement with the intervention.

All participating truck drivers were males. At postintervention, the mean age of participating truck drivers was 43 (range 22-67) years. Less than half of the drivers (10/22, 46%) reported they were aware their workplace had promoted the Facebook page. Of these 10 drivers, most (6/10, 60%) reported they were “satisfied” or “very satisfied” with the Facebook page, but a significant proportion (4/10, 40%) recorded a “neutral” response. Very few drivers reported they “liked” the page to add it to their Facebook feed. Of drivers who responded to the survey question “Where do you get information about your health?”, the proportion of drivers reporting the internet as a source of health information was unchanged between pre- and postintervention (7/44, 15% vs 3/22, 14%, respectively). Furthermore, workplace managers identified a number of reasons for drivers’ lack of engagement with the social media intervention.

Six themes emerged, which represent the key aspects of drivers’, workplace managers’, and workplaces’ engagement with social media as a health promotion intervention.

### Lack of Engagement With Social Media: “There Wouldn’t be One Driver Who Gets on Facebook”

Workplace managers identified drivers’ poor engagement with social media as a key barrier to the effectiveness of the intervention. The managers reported only a small percentage of drivers used social media technologies, such as Facebook, in a private capacity. None of the workplaces used social media specifically, or innovative technology generally, for work purposes. One workplace manager said:

There wouldn’t be one truck driver [in the company] who gets on Facebook. They don’t get on Facebook. I mean, who does get on Facebook?

TWP420

Another workplace manager commented as follows:

I don't think a lot of our drivers are on Facebook. I would say, out of our truck drivers, maybe twenty percent use social media...A lot of them would have mobile phones, for sure. But I'd say the ones that were 'tech savvy' and who would jump on social media, a very small group... A lot of them are over fifty years old.

TWP620

### Impact of Age on Social Media Use: “My Drivers Are the Wrong Age Group”

Workplace managers perceived their predominately middle-aged workforce was the “wrong age” to engage with social media. In a postintervention focus group, drivers discussed this issue, highlighting they were “too old” to be familiar or comfortable with social media. One workplace manager said:

My drivers are the wrong age-group in most cases; they’re not young enough for it, they’re old ‘sticks-in-the-mud’… It’s technology they don’t use… [Most] of the guys are sixty-plus.

TWP120

This was reinforced by a truck driver, who commented:

We’re all sixty years old; we don’t change our thoughts on much.

BTP

### Other Barriers to Social Media Use: “How Would You Get on Facebook?”

There are a number of other barriers to drivers’ use of social media. The theme of “other barriers to social media use” can be organized into 2 subthemes—the prohibitive cost of smartphone technology and drivers’ tendency to confine the use of social media to nonwork-related purposes.

The perceived cost of social media was identified by one workplace manager as a key barrier to the intervention. This manager commented:

And how would you get on Facebook? You'd have to have a [smart]phone... but a lot of fellas wouldn't have those... They cost $700, a lot of drivers wouldn't have $700, you've got to remember that.

TWP420

Another key barrier to the intervention was many drivers confined the use of social media to nonwork-related purposes. In the focus groups, drivers reflected the primary use of social media technologies like Facebook was to connect with family and friends. For example, one manager explained why he did not “like” the page to add it to his Facebook feed:

I keep my Facebook strictly for personal things.

TWP120

### Effect of Workplace Policy on Social Media Use: “We Had a No-Facebook Policy”

A number of workplace managers explained the implementation of the intervention and, specifically, their promotion of the Facebook page was limited by a “no-Facebook policy” at the workplace. Many workplaces had guidelines and policies about social media technologies, such as Facebook, at work and about posting work-related content on such sites. The managers at these workplaces felt the rules limited their capacity to promote the Facebook page to drivers. One workplace manager commented:

No, we didn’t [promote it]… That was when we had a no-Facebook policy… Like a lot of places, there was too much rubbish getting on it, ex-employees and things like that... It becomes a bit of an issue, and there are also privacy issues.

TWP220

The workplace managers identified those drivers who did engage with social media technologies, such as Facebook, were most likely to do so using smartphones. The use of smartphones on the job is illegal while operating trucks. A number of workplace managers reflected on this problem. Additionally, when implementing the intervention, some workplace managers did report attempting to overcome the limitations associated with rules about the use of Facebook and phones in the workplace. One workplace manager said:

I can’t stop them using Facebook in their private time. I’d encourage them to go on and say, ‘Listen, when you’re at home you might like to have a look at it.’

TWP320

### Influence of Others on Social Media Use: “His Wife Runs His Facebook Page for Him”

Drivers were poorly engaged with social media, but their partners and friends were engaged. This was, particularly, true for drivers’ wives, who were identified as being responsible for connecting drivers with social media technologies such as Facebook. For example, one workplace manager said:

There’s one [driver] - his wife runs his Facebook page for him. She just updates the photos of all the grandkids and that sort of stuff. He doesn’t have anything to do with it.

TWP120

Workplace managers reflected on the potential reach of a social media health promotion intervention. This included the potential to reach others in a truck driver’s social circle. One workplace manager commented:

I think [Truckin’ Healthy] sort of worked because I said ‘like’ and then I pressed ‘share’… Even people that I’m associated with outside, that aren’t drivers, liked that… The message is still getting out there… If you want anyone to know anything, just put it on Facebook

TWP520

Another manager commented on the visual immediacy of Facebook for health promotion:

You can see if people like Facebook…

GBFF

### Increased Interest in Social Media Use: “Show Me What’s on There”

The potential of social media in workplace health promotion was identified by many of the workplace managers. Some workplace managers demonstrated a keen interest in learning more about how the technology worked. For example, one workplace manager commented:

My daughter’s got me on Facebook…I knew [the project team] were coming up and I said, “Show me what’s on there”…She said all the trucks have been on…all the pictures of the flood on the farm. I said, ‘You’re joking!’

TWP420

Some workplace managers reflected about new ways of using technology in workplaces, which may be harnessed for health promotion purposes. One workplace manager explained:

We’re rebuilding our Web face and we’re going to do a lot more messaging over [that]. And we’re going to get some Windows tablets for the drivers to use in their trucks, so they’ll have an intranet in their trucks…From there we’ll link all the policies, procedures, all the internal information on the intranet page

TWP320

## Discussion

### Principal Findings

The results of this project reveal a range of barriers to the use of social media as a health promotion intervention in transport industry workplaces. These barriers are (1) truck drivers believe they are the “wrong age” and lack the necessary skills; (2) the cost of smartphone technology is prohibitive; (3) truck drivers confine their use of social media to nonwork-related purposes; and (4) many workplaces have “no Facebook” policies. Other people around truck drivers, particularly partners, do use social media. Many workplace managers identified the potential for social media in workplace health promotion. This section will explore these complex, interrelated concepts in detail.

The key barrier to the use of social media as a health promotion intervention in transport industry workplaces was that drivers do not engage with it for a variety of reasons including demographic constraints and the prohibitive cost of smartphone technology. Further complicating this problem is drivers who do use social media do so primarily for nonwork-related purposes. These issues were not identified during the PAR process undertaken at the beginning of the project. Drivers’ lack of engagement with social media is reflected in other similar studies of health promotion interventions in transport industry workplaces, including those in Australia. For example, Gilson et al [33] reported a 55% uptake in an intervention using smartphone technology to monitor nutrition and physical activity in Australian truck drivers, but concluded, “smartphone technology prohibited a number of drivers from progressing to intervention.” The issue of drivers’ lack of engagement with social media is likely to resolve as smartphone technology becomes more common, in workplaces and wider society, and people of all ages become more accustomed. At present, drivers’ lack of engagement with social media significantly obstructs it as a potential health promotion intervention in transport industry workplaces [42,43].

This project found drivers were poorly engaged with social media, but their partners and friends were engaged. This raises the novel possibility of reaching truck drivers through social media interventions targeted at partners and friends. The importance of family and peer support for positive health outcomes has been demonstrated by other studies on health interventions in blue-collar workplaces [44-48]. This idea was not explored in this research, but it identifies an important area for future research. This research suggests if social media interventions in older male populations are to be effective, it is important to consider—and, where possible, utilize—their family context.

Another key barrier to the effectiveness of social media in health promotion interventions in transport industry workplaces is workplace policy. Many workplaces had “no-Facebook” policies, implemented because of drivers’ inappropriate use of smartphones at work. The literature and popular media reveal these as common problems. For example, there are numerous reports of drivers’ dismissals after breaches of workplace social media policies [7,49,50] and of serious road accidents caused by drivers’ irresponsible use of smartphones while operating heavy vehicles [51-53]. The use of any mobile phone while driving is illegal in Australia. A potential solution to this barrier, as suggested by one of the workplace managers during an informal “corridor conversation,” is to implement social media health promotion interventions in which truck drivers are encouraged or, perhaps, incentivized in their spare time. This may be problematic considering issues such as long working hours, fatigue, and limited family time, anecdotally reported by many drivers.

A number of other barriers have been discussed in the literature with regard to social media in workplace health promotion interventions. These barriers include a lack of knowledge and understanding of social media among workplace managers and workplace managers’ failure to accept new ways of thinking related to the use of social media [54]. These barriers were not apparent in this project. Many of the workplace managers in this research demonstrated a keen interest in social media. Some had ideas about how social media could be used in their workplace in future, for health promotion and other applications. The intervention implemented was suggested by one of the workplace managers, demonstrating workplace managers can play an active role in developing local social media strategies to engage their “hard-to-reach” workforce in health promotion.

Despite considerable barriers, this project and the literature suggest there is great potential for social media in health promotion interventions in transport industry workplaces. Much more needs to be known about how to best use social media to engage with the target audience and, particularly, “hard-to-reach” groups such as truck drivers [8]. Hudson and Hall [55] recommended creating a “trial period” prior to the implementation of a social media intervention. The aim of a trial period is to develop an understanding of the target audience and build a “credible and engaging content strategy” focusing on the audience’s unique interests and needs [55].

The contextualization of interventions in this way is a fundamental aspect of the PAR process, which underpinned this project [37]. The findings paper for this project (published elsewhere) demonstrates the contextualization of interventions is pivotal to achieving workplace culture change and improvements in health knowledge and behavior in the participating truck drivers. This is an effect observed in other health promotion interventions in blue-collar workplaces [47,48,55-58]. Future projects should seek to engage participants to a greater extent in the design of a social media health promotion intervention. This would have the effect of fostering a more integrated Web-based network beyond simply “joining a Facebook group,” a key aspect of engaging “hard-to-reach” groups in social media health promotion interventions [59,60].

In addition to the contextualization of a social media workplace health promotion intervention, the contextualization of strategies to support and deliver this intervention needs to be considered. In this project, workplace managers used posters displayed in depots to advertise the Facebook page. Some mentioned the page in toolbox talks with drivers. Other studies using social media to engage “hard-to-reach” groups in health promotion interventions have used more rigorous advertising, which is integrated with other interventions, including posters, personal health messages, and public announcements [59]. This delivers a more cohesive intervention strategy and provides opportunities to “piggyback” the advertisement of less-popular interventions on more-popular ones with higher levels of engagement. Other recent studies seeking to engage “hard-to-reach” groups, such as blue-collar workers, using social media health promotion interventions, deliver interventions through a range of different channels, including Facebook, Twitter, Skype, Instagram, and other popular apps [59,61-64]. This multipronged approach significantly increases the reach of the intervention and provides additional opportunities to tailor strategies to each workplace’s unique context.

### Limitations and Future Research

There are a number of limitations to this project. First, the convenience sample of truck drivers may create self-selection bias. Second, the project relied on the self-reporting of data in interview and focus groups, which may have resulted in socially desirable responses. Lastly, the small sample size and highly contextual nature of the social media intervention limits the generalizability of findings. However, other organizations with a small-to-medium sized, blue-collar, mobile workforce may find them of relevance.

Future projects investigating social media in “hard-to-reach” workplaces should consider a larger sample from a wider demographic, including rural and regional workplaces, to improve the generalizability. Consideration should be given to improving engagement with social media interventions by integrating these with other interventions that do not use social media, by using a variety of social media technologies and engaging workplace managers, workers, and their families in the intervention design. Furthermore, future workplace health promotion interventions should consider reaching truck drivers through social media interventions targeted at partners and friends.

### Conclusions

Transport industry workplaces, in Australia and other countries, are increasingly technology-centric, and truck drivers are engaging with social media in their workplaces on a day-to-day basis. This project found that truck drivers participating in a workplace health promotion intervention involving Facebook were generally poorly engaged because of their older age, lack of skills with social media, the cost of smartphone technology, or their workplace “no Facebook” policy. Truck drivers’ family and friends were engaged with this intervention, which suggests if social media interventions in older male populations are to be effective, it is important to consider—and, where possible, utilize—their family context. The findings showed a high level of engagement from workplace managers in developing local social media strategies, which attests to the potentially important role of managers in engaging “hard-to-reach” workforce in health promotion. Therefore, it is important to gain greater insights into social media in workplace health promotion interventions and understand the enablers and inhibitors to potential use in the transport industry.

Despite some barriers evident in truck drivers, it is important to explore the potential of social media in workplace health promotion interventions in the transport industry. Much more needs to be known about how to best use social media to engage with “hard-to-reach” groups such as truck drivers. This can be achieved by methods of inquiry, such as PAR, which allow interventions to fit the sociocultural context of workplaces. Strategies for the contextualization include the involvement of occupational groups in the design of social media workplace health promotion interventions and strategies to support and deliver interventions. The context of workers themselves, including their age and familiarity with social media, and work, workplace, and family context is important to consider in this process. The “context matters” because it enables the development of more nuanced, integrated, and extensive Web-based networks, key aspects of engaging “hard-to-reach” groups in social media interventions for health promotion.

## Abbreviations

PAR: Participatory Action Research
